# Supplementary material for: The Effects of Omega-3 Supplementation on Depression in Adults with Cardiometabolic Disease: A Systematic Review of Randomised Control Trials
Source: Nutrients. 2022 Apr 27;14(9):1827. doi: 10.3390/nu14091827 (PMC9101438; doi:10.3390/nu14091827)
Supplement: Supplementary file 1 [file nutrients-14-01827-s001.zip › nutrients-1682804-supplementary.pdf]

Supplementary Material:

Table S1: Collated risk of bias grading for each domain for included studies.

Trial	Randomisation		Blinding		Attrition bias	Reporting bias	Adherence to treatment	Missing outcome data	Overall risk of bias score
	Random sequence generation	Allocation concealment	Blinding of participants and personnel	Blinding of outcome assessment	Incomplete outcome data	Selective reporting	Adherence to treatment		
**Bot et al. 2010**	**High**	**Low**	**Low**	**High**	**Low**	**Moderate**	**Low**	**Low**	**High**
**Carney et al. 2009**	**Low**	**Low**	**Low**	**Moderate**	**Low**	**High**	**Low**	**Low**	**Moderate**
**Chang et al. 2019**	**Moderate**	**Moderate**	**High**	**High**	**Low**	**High**	**Moderate**	**Low**	**High**
**Jiang et al. 2018**	**Low**	**Low**	**Low**	**Low**	**Moderate**	**Low**	**Moderate**	**Low**	**Moderate**
**Mazaherioun et al. 2018**	**Low**	**Low**	**Low**	**Low**	**Low**	**Moderate**	**Low**	**Low**	**Low**
**Mazereeuw et al. 2016**	**Moderate**	**Low**	**Low**	**Low**	**Low**	**Low**	**Low**	**Low**	**Low**
**Zimmer et al. 2013**	**Low**	**Low**	**Low**	**Low**	**Low**	**Moderate**	**Low**	**Low**	**Low**

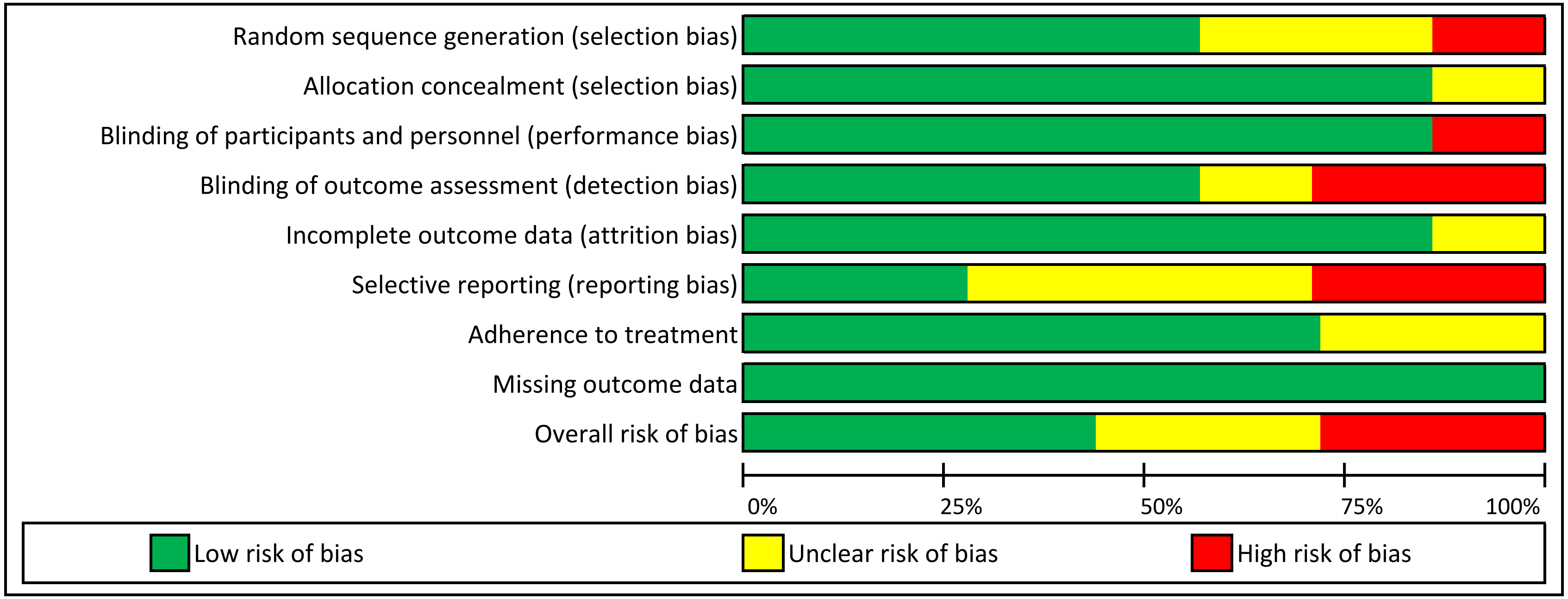

Figure S1: Risk of bias graph expressed as a percentage across included studies based on reviewers' judgement.

Table S2: Recommendations to improve methodological rigour in Omega-3 clinical trials

Area for improvement	Recommendation
**Baseline differences**	▪ Assess and adjust for baseline differences between habitual Omega-3 and Omega-6 diet and supplement intake<br>▪ Assess and adjust for baseline difference in Omega-3 blood levels during randomisation
**Inclusion/Exclusion criteria**	▪ Exclude participants on medications that can interact with Omega-3 absorption<br>▪ Include populations with medical diagnosis of MDD only<br>▪ Ensure appropriate prior piloting of intervention to describe sufficient numbers needed to observe an effect
**Methodological**	▪ Clearly justify total Omega-3 dosage, formulation (EPA/DHA ratio) and frequency<br>▪ Define a suitable intervention period (weeks) needed to demonstrate sufficient Omega-3 bioaccumulation and allow for Omega-3 effects on depression symptoms to occur<br>▪ Do not use a fatty acid with suspected or confirmed similar cardiovascular or mental health benefits as placebo<br>▪ Opt for secure and auditable database entry and management systems (e.g. REDCap) over traditional data collection methods to maximise data integrity and enable real-time data monitoring<br>▪ Ensure blinding of outcome assessment to enable triple blinding
**Analysis**	▪ Publish pre-specified statistical analysis plan in the clinical trials registry and report deviations where applicable

File S1. Search Strategy

CINAHL

[Population]

S1 (MH “Depression”)

S2 (MH “Depression, Reactive”)

S3 (MH “Dysthymic Disorder”)

S4 (MH “Affective Disorders”)

S5 (MH “Affective Symptoms”)

S6 (depress* or dysthymi* or “adjustment disorder*” or “affective disorder*” or

“affective

symptom*” or “mood disorder*”)

S7 (S1 or S2 or S3 or S4 or S5 or S6 or S7)

[Intervention]

S8 (MH “FISH OILS”)

S9 (MH “FATTY ACIDS, OMEGA-3”)

S10 (MH “DOCOSAHEXAENOIC ACIDS”)

S11 (MH “EICOSAPENTAENOIC ACID”)

S12 (AB ((DHA or Docosahex* or Eicosapent* or EPA or “fatty acid*” or fish* or

linolenic or

omega-3 or n-3 or w-3 or PUFA* or “cod liver oil” or “cod-liver oil”)) OR TI ((DHA or

Docosahex* or Eicosapent* or EPA or “fatty acid*” or fish* or linolenic or omega3 or

n-3 or

w-3 or PUFA* or “cod liver oil” or “cod-liver oil” OR)))

S13 (S8 or S9 or S10 or S11 or s12)

*RCT Filter+ S14 (MH “Clinical Trials+”)

S15 (PT Clinical trial) S16 (TX clini* n-3 (trial* or study or studies)) S17 (TX ((singl*

N1 blind*)

or (singl* N1 mask*)) or TX ((doubl* N1 blind*) or (doubl* N1 mask*)) or TX ((tripl* N1

blind*) or (tripl* N1 mask*)))

S18 (TX random* n-3 control*)

S19 (MH “Random Assignment”) 46

S20 (TX random and (allocat* or assign*))

S21 (TX placebo*)

S22 (TX (waitlist* or (wait* and list*)) and (control* or group))

S23 (TX “treatment as usual” or TI TAU or AB TAU)

S24 (TX (control* n-3 (trial* or study or studies or group*)))

S25 (MH “Quantitative Studies”)

S26 (S14 or S15 or S16 or S17 or S18 or S19 or S20 or S21 or S22 or S23 or S24 or

S25)

S27 (S7 and S13 and s26)

MEDLINE

1 exp Depression/

2 exp Dysthymic Disorder/

3 exp Mood Disorders/

4 exp Affective Symptoms/

5 (depress* or dysthymi* or adjustment disorder* or affective disorder* or affective symptom* or mood disorder*).mp. [mp=title, abstract, original title, name of substance word, subject heading word, floating sub-heading word, keyword heading word, organism supplementary concept word, protocol supplementary concept word, rare disease supplementary concept word, unique identifier, synonyms]

6 1 or 2 or 3 or 4 or 5

7 exp Fish Oils/

8 exp Fatty Acids/

9 exp Fatty Acids, Omega-3/

10 exp Docosahexaenoic Acids/

11 exp Eicosapentaenoic Acid/

12 (DHA or Docosahex* or Eicosapent* or EPA or fatty acid* or fish* or linolenic* or linolenic acid or omega-3 or n-3 or w-3 or PUFA* or cod liver oil or cod-liver oil).mp. [mp=title, abstract, original title, name of substance word, subject heading word, floating sub-heading word, keyword heading word, organism supplementary concept word, protocol supplementary concept word, rare disease supplementary concept word, unique identifier, synonyms]

13 7 or 8 or 9 or 10 or 11 or 12

14 random* control* trial.mp.

15 6 and 13 and 14

Cochrane Library

#1 - MeSH descriptor: [Depression] explode all trees

#2 - MeSH descriptor: [Depressive Disorder] explode all trees

#3 - MeSH descriptor: [Fatty Acids, Omega-3] explodes all trees and with qualifier(s): [adverse effects – AE, therapeutic use – TU]

#4 - (#1 OR #2) AND #3

*** Search strategy is an adapted version of the search strategy used in Appleton et al. 2016 Cochrane review's "ω-3 Fatty acids for major depressive disorder in adults: an abridged Cochrane review" Appleton KM, Sallis HM, Perry R, Ness AR, Churchill R. ω-3 Fatty acids for major depressive disorder in adults: an abridged Cochrane review [published correction appears in BMJ Open. 2017 Jan 16;7(1):e010172corr1]. BMJ Open. 2016;6(3):e010172. Published 2016 Mar 2. doi:10.1136/bmjopen-2015- 010172 ***
